# Trends in sepsis mortality over time in randomised sepsis trials: a systematic literature review and meta-analysis of mortality in the control arm, 2002–2016

**DOI:** 10.1186/s13054-019-2528-0

**Published:** 2019-07-03

**Authors:** Robert Luhr, Yang Cao, Bo Söderquist, Sara Cajander

**Affiliations:** 1 School of Medical Sciences, Faculty of Medicine and Health, Örebro University, 70182 Örebro, Sweden; 2 Clinical Epidemiology and Biostatistics, School of Medical Sciences, Örebro University, 70182 Örebro, Sweden; 3 Unit of Biostatistics, Institute of Environmental Medicine, Karolinska Institutet, 17177 Stockholm, Sweden; 4 Department of Infectious Diseases, Örebro University Hospital, 70182 Örebro, Sweden

**Keywords:** Severe sepsis, Septic shock, Mortality, Randomised controlled trial, Meta-analysis

## Abstract

**Background:**

Epidemiologic data have shown an increasing incidence and declining mortality rate in sepsis. However, confounding effects due to differences in disease classification might have contributed to these trends.

To assess if a declining mortality over time could be supported by data derived from high-quality prospective studies, we performed a meta-analysis using data from randomised controlled trials (RCTs) on sepsis. The primary aim was to assess whether the mortality in sepsis trials has changed over time. The secondary aim was to investigate how many of the included trials could show efficacy of the studied intervention regarding 28-day mortality.

**Methods:**

We searched PubMed for RCTs enrolling patients with severe sepsis and septic shock, published between 2002 and 2016. The included trials were assessed for quality and sorted by date of first inclusion. A meta-analysis was performed to synthesise data from the individual sepsis trials.

**Results:**

Of 418 eligible articles, 44 RCTs on sepsis were included in the analysis, enrolling 13,315 patients in the usual care arm between 1991 and 2013. In this time period, mortality decreased by 0.42% annually (*p* = 0.04) to give a total decline of 9.24%. In subgroup analyses with adjustments for APACHE II, SAPS II and SOFA scores, the observed time trend was not significant (*p* = 0.45, 0.23 and 0.98 respectively). Only four of the included trials showed any efficacy with regard to mortality.

**Conclusions:**

Data from RCTs show a declining trend in 28-day mortality in severe sepsis and septic shock patients during the years from 1991 to 2013. However, when controlling for severity at study inclusion, there was no significant change in mortality over time. The number of trials presenting new treatment options was low.

**Trial registration:**

PROSPERO CRD42018091100. Registered 27 August 2018.

**Electronic supplementary material:**

The online version of this article (10.1186/s13054-019-2528-0) contains supplementary material, which is available to authorized users.

## Background

Sepsis is a potentially life-threatening condition which is estimated to affect over 30 million people worldwide each year [1]. Recent data suggest that sepsis contributes to between one third and half of all hospital deaths in the USA and accounts for a significant part of the overall costs of healthcare [2–4]. The incidence of severe sepsis and septic shock in developed countries has been reported as increasing steadily over recent decades; for example, during 2004–2009, the incidence of severe sepsis increased from 300 to 1031 cases per 100,000 population in the USA [5]. Conversely, several reports from different geographical areas have shown a sequential decrease in sepsis mortality over time. In a large study of European ICU patients over time, the odds of ICU mortality in sepsis patients were lower in 2012 versus 2002 [6]. Similar to this, a study from Germany based on retrospectively collected administrative data for 2007–2013 found a declining mortality trend, although the incidence was increasing [7]. A similar trend was also reported for sepsis patients treated in intensive care units in Australia and New Zealand during 2000–2013 [8], though the authors also identified a substantial variation in mortality risk according to the classification of the diagnosis of sepsis, implying a potential risk that reported temporal trends in sepsis mortality based on administrative data may be biased by misclassification. Results might have been influenced by differences due to increased awareness of the disease or changes in diagnosis coding practices over time, as well as incorrect coding due to retrospectively derived data [9]. Extracting clinical data from electronic databases seems to be the most unbiased way to study trends in hospital mortality [10–14]. With this approach, previous assumptions of declining in-hospital sepsis mortality in the USA were shown to be partly confounded by increased disease awareness and sepsis diagnosis coding [10]. However, the accessibility to large sets of clinical data from electronic health records is often limited in many countries outside the USA and only hospital mortality can be studied.

In order to study trends of mortality in patients with severe sepsis with reduced risk of classification bias, Stevenson et al. assessed the trends of sepsis mortality based on data from randomised controlled trials (RCTs) [15]. They found a decrease in sepsis mortality between 1991 and 2009, consistent with administrative data and supported the use of administrative data to monitor mortality trends in patients with severe sepsis. As this was the first meta-analysis performed with this research question, we wanted to assess if the results were reproducible in a prospectively designed meta-analysis of high-quality sepsis trials, published during a different time interval.

Accordingly, we hypothesised that a trend in the mortality of patients enrolled in the usual care arm of interventional high-quality sepsis studies published between 2002 and 2016 should be concordant with the previously reported trends from RCT data where quality assessments of the included studies had not been performed.

The primary aim of this study was to investigate whether the reported 28-day mortality of patients with severe sepsis and septic shock, included as controls in randomised controlled trials, has declined over time. The secondary aim was to assess how many of the included trials could show any beneficial effect of the studied intervention with regard to 28-day mortality.

According to international guidelines, there is currently no approved sepsis-specific drug or intervention [16]. In addition to investigating any apparent temporal trends in mortality, we therefore also sought to explore how many studied interventions have shown efficacy in terms of mortality.

The study was registered in the International Prospective Register of Systematic Reviews (https://www.crd.york.ac.uk/PROSPERO/) and given number CRD42018091100. The Preferred Reporting Items for Systematic Reviews and Meta-Analyses (PRISMA) Statement and the Meta-Analysis of Observational Studies in Epidemiology (MOOSE) Statement were referred to for methodological guidance [17].

## Materials and methods

### Literature search

The PubMed database was searched for RCTs of patients with severe sepsis and/or septic shock, published between 2002 and 2016. The full search sequence is detailed in Additional file 2: Table S1.

The research question was devised in accordance with the Patient, Intervention, Comparison and Outcome (PICO) model: P, patients aged 16 years or older presenting with severe sepsis or septic shock; I, all treatments of severe sepsis and septic shock regardless of length of treatment and hospital setting; C, usual care; and O, mortality at 28 ± 2 days.

Two independent investigators (RL and SC) reviewed abstracts in order to select potentially eligible studies according to pre-defined inclusion criteria. All selected studies were subsequently evaluated for quality assessment using the Jadad score [18].

Studies were excluded if they (i) enrolled fewer than 50 patients in the control arm, since small trials may be more likely to have biased estimates, (ii) did not report mortality by day 28 (± 2), (iii) were considered to be of poor quality (Jadad score < 3), (iv) used data from other RCTs or (v) were written in a language other than English. To minimise the risk of the results being influenced by the difference in disease classification, only trials using the first and second international consensus definition of sepsis were included [19].

### Data extraction

The data extraction required for meta-analysis included the following information: (1) mortality at 28 ± 2 days, (2) number of patients enrolled in the usual care group, (3) number of patients in the intervention group, (4) whether or not the studied intervention had any beneficial effect on mortality, (5) age of the patients, (6) severity of condition at inclusion (defined as the average value of the APACHE II, SAPS II or SOFA score), (7) country, (8) author, (9) year of publication and (10) enrolment period.

### Statistical analysis

To analyse temporal trends in severe sepsis and septic shock mortality, we performed a meta-regression analysis of the included trials pooled by year of first patient inclusion [20]. The temporal trend of mortality was modelled as:

$$ {\mathrm{mortality}}_i={\beta}_0+{\beta}_1\times {\mathrm{year}}_i+\varepsilon $$

where mortality_*i*_ is the mortality in the year *i* and *β*_1_ (slope) is the estimated temporal effect size per year on the pooled mortality. The same model was used to evaluate trends in age and disease severity. To reveal the effects of potential confounding factors, we also performed sensitivity analyses that included the average age of the patients as a dichotomous variable (< 65 vs. ≥ 65 years) in the meta-regression model.

The Higgins *I*^2^ was calculated to evaluate the statistical heterogeneity across the studies [21], with *I*^2^ > 30% considered as at least moderate heterogeneity. The pooled relative risk (RR) with corresponding 95% confidence interval (CI) was presented based on the random effects model. To investigate differences between the efficacy of different types of intervention, the trials were divided into eight subgroups and, accordingly, the RR were pooled within each subgroup.

The statistical analyses were performed in version 15.1 of Stata (Stata Corp., College Station, TX, USA). All statistical tests were two-sided. Overall combined RRs with 95% CIs and *p* values were calculated. Statistical significance was defined as a 95% CI excluding 0 or a RR excluding 1 and two-sided *p <* 0.05, except where otherwise specified.

### Ethical considerations

This was a systematic review of previously published data from trials which had all been ethically approved before commencement, so no additional ethical approval was considered necessary.

## Results

### Study characteristics

**Fig. 1:**
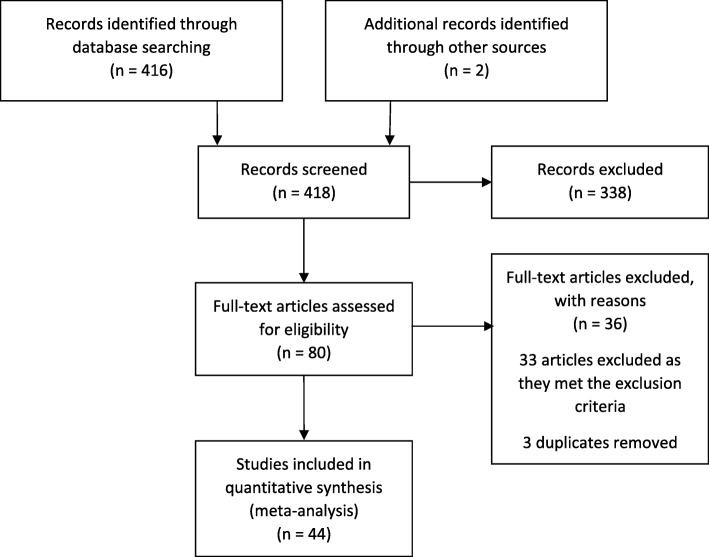
Flowchart showing the selection process of articles eligible for meta-analysis

Of the 418 publications reviewed, 44 were included in the final analysis (Fig. 1 and Additional file 3: Table S2). These enrolled a total of 27,733 patients (13,315 patients in the usual care arm and 14,418 in the intervention arm) from 55 countries and 6 continents (Additional file 3: Table S2). Sorting the studies by date of first patient inclusion generated a time span of 22 years (1991–2013). Among the patients enrolled in the usual care arm, mean age was 62.7 (± 4.2) years, mean Acute Physiology and Chronic Health Evaluation (APACHE) II score was 22.4 (± 3.7), mean Simplified Acute Physiology Score (SAPS) II score was 52.9 (± 4.5) and mean Sequential Organ Failure Assessment (SOFA) score was 8.4 (± 2.1) (Table 1).

**Table 1:** Geographic distribution and quality of the 44 included trials

	*n* (%)
Multicentre trials	41 (93)
Trial country	
France	5 (11)
Germany	5 (11)
Italy	3 (7)
UK	2 (5)
USA	2 (5)
Australia	1 (2)
China	1 (2)
India	1 (2)
Singapore	1 (2)
Zambia	1 (2)
N/A	2 (5)
Multinational	20 (45)
Jadad score	
5	19 (43)
4	8 (18)
3	17 (38)

### Temporal trend of mortality

**Fig. 2:**
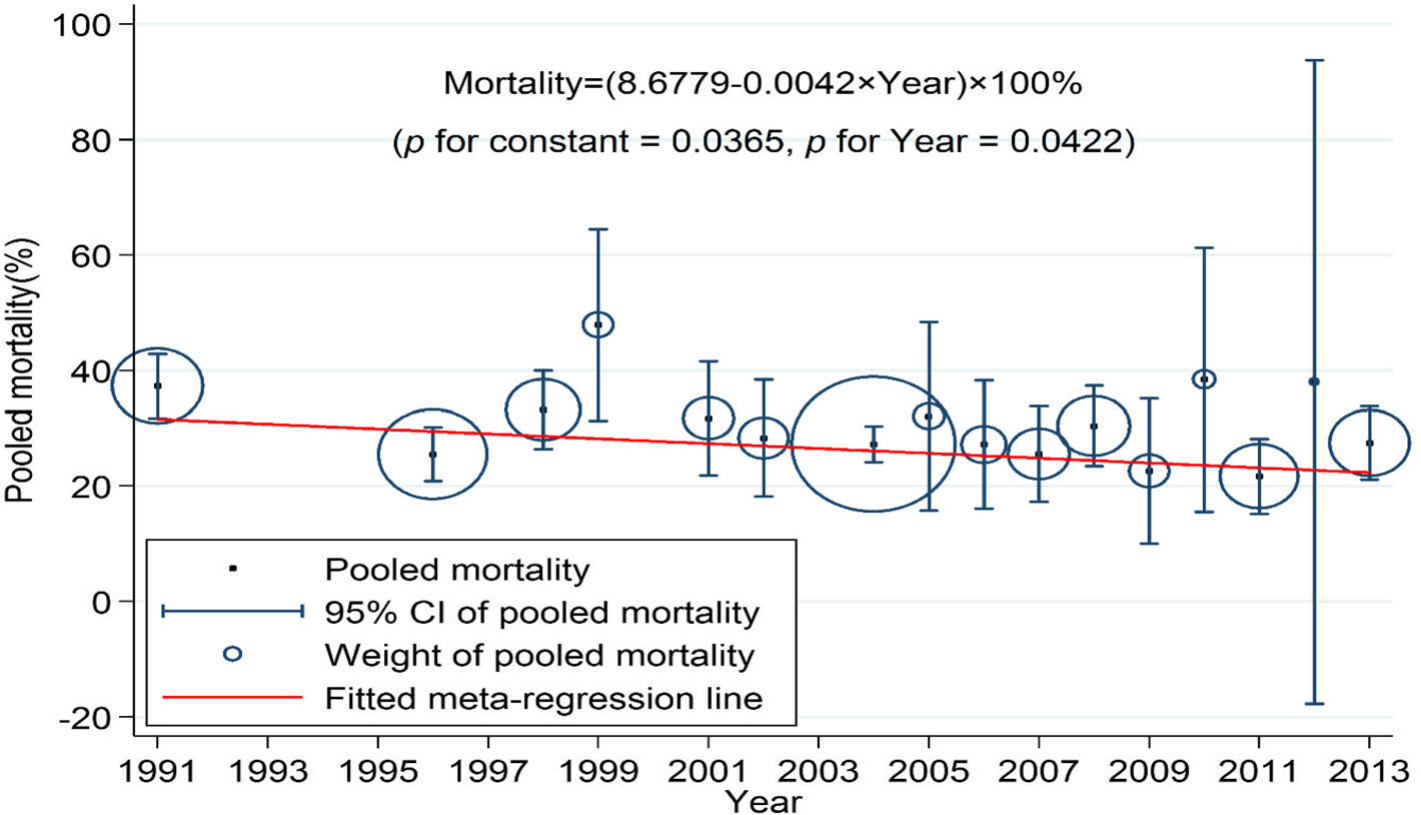
Regression analysis of the included sepsis trials regarding 28-day mortality. Studies are pooled by year of first patient enrolment. The size of the circle denotes the weight of the pooled mortality for each year

The meta-regression analysis showed a decrease in 28-day mortality of 0.42% per year (*p* = 0.04) between 1991 and 2013 (Fig. 2), giving a total decrease of 9.24% during the studied time period.

### Subgroup analysis

A subgroup analysis with mean age modelled on a dichotomous variable (< 65 vs. ≥ 65 years) revealed that mortality was 8% higher in studies including patients with a mean age ≥ 65 years. Meta-regression of the mortality trend with adjustment for dichotomous age (< 65 vs. ≥ 65 years) still demonstrated a significant decline of 0.57% annually (95% CI − 0.010 to − 0.001; *p* = 0.01). APACHE II, SAPS II and SOFA scores on study inclusion were reported in 28, 14 and 24 studies, respectively (Additional file 3: Table S2). During the studied time period, there was a significant decline of 0.27 points annually in APACHE II score (*p* = 0.03) (Additional file 1: Figure S1). There was no trend of significance over time in the SAPS II and SOFA scores (*p* = 0.78 and *p* = 0.37, respectively). When adjusting the meta-regression analysis of the mortality over time for the three severity scores, the trend was no longer significant (adjusted *p* values for APACHE II, SAPS II and SOFA were 0.45, 0.23 and 0.98, respectively.

### Efficacy of study interventions

**Fig. 3:**
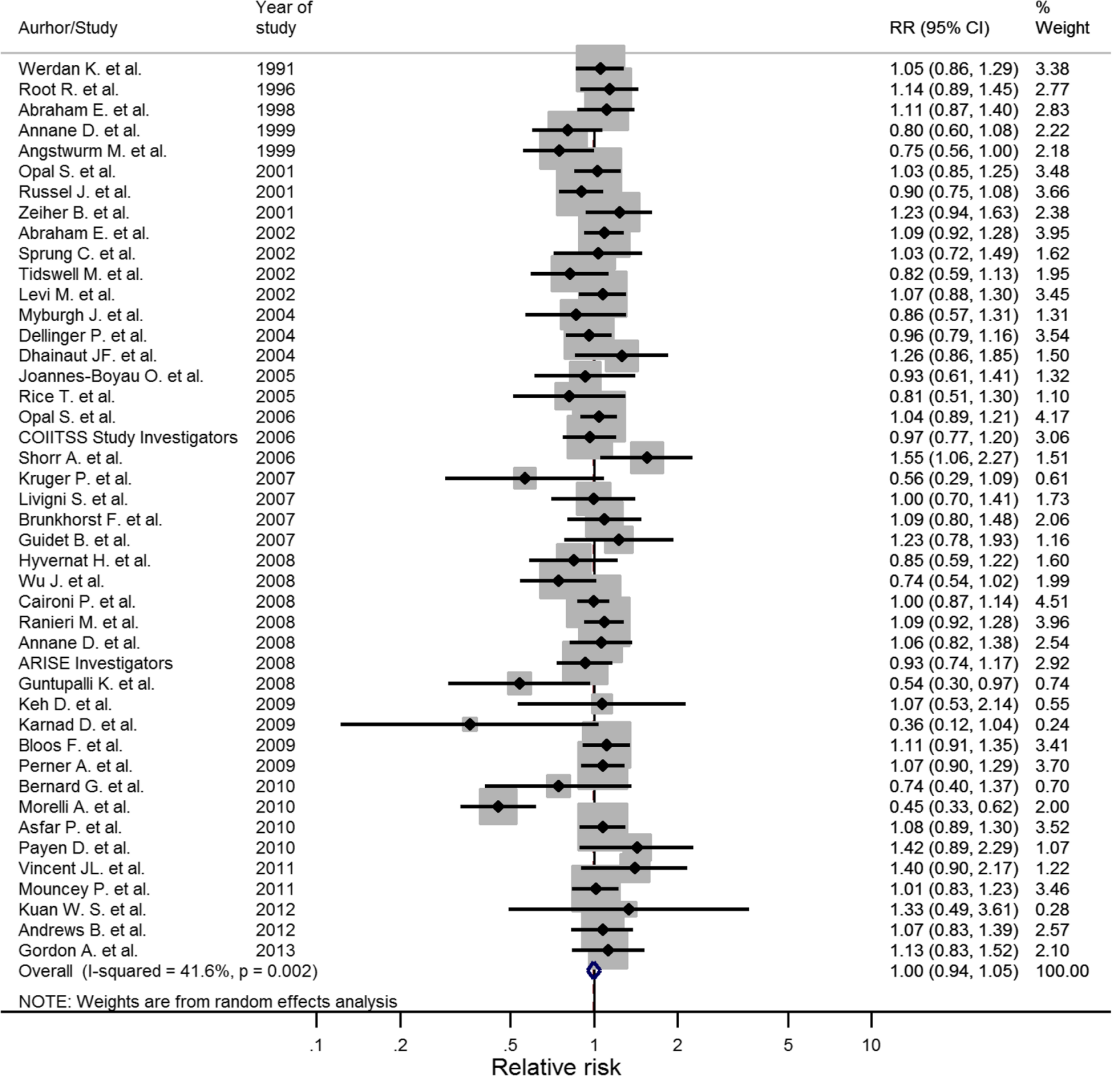
Forest plot displaying the relative mortality risks (RRs) of the interventions studied in the included sepsis trials. The individual points denote the RR of each study and the lines either side the 95% confidence intervals. The vertical line denotes the null effect

The overall relative risk of mortality in the 44 included trials was 1.00 (95% CI 0.94 to 1.05). Four of the 44 trials showed the efficacy of the studied intervention with regard to 28-day mortality (Fig. 3). These four studies targeted the following interventions: selenium, talactoferrin, esmolol and ulinastatin [22–25]. A subgroup analysis of intervention efficacy, grouped by type of intervention, revealed a significantly higher relative mortality risk for the intervention in trials studying modulation of coagulation and inflammation [26–31] (RR = 1.113; 95% CI 1.019 to 1.216; *p* = 0.02; Table 2). This subgroup analysis included Higgin’s *I*^2^ test to evaluate the potential heterogeneity in the selected subgroups. Three of the examined subgroups showed an *I*^2^ value over 30%. Only one group, the group called “Other interventions”, did however show any heterogeneity of significance (*p* = < 0.001).

**Table 2:** Subgroup analysis for relative risk (RR) of intervention vs. control

Type of study	Number of studies	Combined RR (95% CI)	*p* value	*I*^2^**	*p* value for *I*^2^
Early goal-directed therapy	3	0.985 (0.860, 1.127)	0.824	0.0%	0.686
Hemofiltration	2	1.149 (0.840, 1.573)	0.384	44.6%	0.179
Immunomodulation	18	0.996 (0.931, 1.066)	0.917	32.5%	0.090
Modulation of coagulation and inflammation	6	1.113 (1.019, 1.216)	0.018	0.07%	0.600
Other interventions	7	0.912 (0.751, 1.106)*	0.531	80.3%	< 0.001
Plasmapheresis	1	0.995 (0.702, 1.410)	0.978		
Vasoactive drugs	4	0.916 (0.804, 1.044)	0.189	0.0%	0.447
Fluid resuscitation	3	1.019 (0.897, 1.159)	0.769	0.0%	0.593

*Random effects model was used

**I^2^ measures the statistical heterogeneity across the studies

## Discussion

In this study, we performed a meta-regression analysis of data derived from RCTs published between 2002 and 2016 (inclusion start date from 1991 to 2013) after a systematic literature search of studies including severe sepsis and septic shock. Our primary aim was to study the mortality trend of patients enrolled in the control arm. We found an annual decrease of 0.42% in the 28-day mortality among all patients enrolled as controls. Interestingly, the mortality trend was still a significantly decreasing one after adjustments for age and was found to be more pronounced in studies enrolling patients with a mean age ≥ 65 years. Severity scores were not adjusted for in our primary meta-regression analysis as different severity scores were used between the studies. However, when performing subgroup analyses with adjustments for APACHE II, SAPS II and SOFA scores on study inclusion, the mortality trend was no longer significantly decreasing over time. Consequently, the observed sepsis mortality decline in the control arm of RCTs could in part be explained by the inclusion of patients with a lower predicated mortality over time.

In the similar study from 2014 by Stevenson et al., also using data from sepsis patients included as controls of RCTs, a declining mortality trend by 3.0% annually was demonstrated [15]. The results of the unadjusted analysis in the present study support their results of a declining mortality trend in the control arm of sepsis trials, although an important difference was found as they demonstrated that the declining sepsis mortality was not confounded by changes in disease severity at inclusion. This might be due to differences in the inclusion and exclusion criteria of the studies entered in the meta-regression analysis. In particular, we included trials during a later time span and included studies enrolling patients who were ≥ 16 years old and excluded studies with poor quality. Additionally, Stevenson et al. performed adjustments of the meta-regression from estimations of predicted mortality derived from different severity assessment scales while we performed separate subgroup analyses. This might include errors as SAPS II and APACHE II are shown to differ in the prediction of hospital mortality [32].

Another important issue that should be discussed in relation to the study by Stevenson et al. is their final conclusion stating that a declining sepsis mortality over time in the control arm from RCTs supports the use of administrative data to monitor trends in sepsis mortality. In our opinion, this may be questioned as several studies comparing clinical data with administrative claims data have shown that the decreases in sepsis mortality over time shown by administrative data are overestimated [10–13].

The so-called Will Rogers phenomenon explains reduced disease-related mortality as a result of increased disease awareness and a higher proportion of less severely ill patients [33]. Indeed, the Will Rogers phenomenon could also be relevant in the current study due to changes in triage screening systems and to increased focus on early goal-directed treatment; as shown in a retrospective study on trends in usual care of septic shock patients between 2003 and 2013, time to initiation of antibiotics and fluid therapy in the emergency department was shown to be significantly shortened over time [34]. Several retrospective studies have demonstrated a clear association between early administration of antibiotic treatment and reduced sepsis mortality [35–37]. The results of these studies have led to the implementation of scoring systems for early identification of patients with suspected sepsis [38] and evidence-based treatment bundles. In 2002, the Society of Critical Care Medicine and the European Society of Intensive Care Medicine implemented a new set of guidelines, the Surviving Sepsis Campaign, as an aid in the treatment of patients with severe sepsis and septic shock [39]. Medical centres following these guidelines to a higher degree have been shown to have a lower mortality in septic patients [40].

As a secondary aim of this study, we assessed the intervention effect on 28-day mortality. Overall, we found no efficacy regarding 28-day mortality. Four of the 44 included trials showed a beneficial effect of the studied intervention on mortality [22–25]. However, two of these interventions, which used selenium and talactoferrin respectively, failed to demonstrate any benefit when replicated in larger studies with multicentre settings [41, 42]. The other two treatment strategies with proven efficacy (esmolol and ulinastatin) [24, 25] have not yet been implemented in the guidelines for standard care of sepsis, as their clinical effect needs further evaluation, although ulinastatin has been described as a promising novel treatment option [43]. Our subgroup analysis of the interventions and their effect on mortality revealed a significant increase in relative risk in the group of interventions categorised as “modulation of coagulation and inflammation”. All these trials investigated the effect of activated recombinant protein C (Xigris®). This result supports the reported risks of this specific treatment which led to its discontinuation [30].

As already established, sepsis is largely a heterogeneous syndrome. This diversity extends both to administrative aspects, leading to risks of classification errors, and to the pathobiology constituting the disease, creating a great variance in the presentation of the ailment based on properties of the pathogen and the host alike. The heterogeneity of patients included in RCTs investigating interventions for sepsis is probably an important reason for a large number of trials that have failed to prove efficacy. Although many post hoc analyses of large RCTs have been performed to control for this, including stratification to identify subgroups of patients who may benefit from the interventions that failed to prove efficacy, individualised treatments have not been implemented in clinical care [44]. Precision medicine with treatments guided by specific geno- or phenotypes seems to be a promising future approach to find interventions with efficacy in certain subgroups [45–47] but require molecular diagnostics with short turnaround to clinicians. Interestingly, four novel subtypes of sepsis based on routinely available clinical data, have recently been shown to predict for differences in treatment responses of previous sepsis trials [48].

In order to address the difficulties caused by the heterogeneity of the disease, in 2016, the Society of Critical Care Medicine and the European Society of Intensive Care Medicine presented the third international consensus definition for sepsis and septic shock [49]. This aims to minimise the risk of misclassification and ease the management of the condition by presenting a more restricted definition of sepsis. This restricted definition may however increase the false negatives, as shown by Fang et al. [50]. It remains to be seen how this will affect heterogeneity in future studies, but hopefully this will ease the process of studying the disease, leading to a better understanding of the condition.

In recent decades, even though only a few specific interventions have shown some effect on mortality, progress has been made and embraced in the shaping of international guidelines. Several studies have raised a warning flag regarding excessive fluid therapy [51, 52], one of which reported a correlation between the amount of administered fluids and death [52], leading current guidelines to suggest a more restrictive approach [16]. Moreover, in 2012, Perner et al. reported an increase in adverse events while treating patients with septic shock with hydroxyethyl starch (a synthetic colloid) [53], with the result that it is no longer used in clinical management. Current guidelines advocate fluid resuscitation to be primarily constituted by crystalloids and, if needed, addition of albumin rather than any synthetic colloid [16]. Furthermore, improvements in sepsis survival in ICUs may be related to the use of a protective-ventilation strategy in patients with sepsis-induced acute respiratory distress syndrome, which correlates with increased survival [54].

Our study has several strengths. First, as this meta-analysis included over 13,000 patients enrolled in the control group in 44 trials from 55 countries and 6 continents, the reported results do not seem to be confined to a single part of the world. Moreover, all studies considered for inclusion were assessed for quality and excluded if they did not meet the pre-defined quality criteria. Lastly, as we used 28-day mortality ± 2 days as an inclusion criterion, the risk of missing important papers on the matter was reduced.

This study also has several important limitations. First, we only used one database to conduct our search and so may have left some studies out of our analysis. Second, the exclusion of trials published in languages other than English may have limited the findings of this meta-analysis to Western countries. Third, our choice to exclude trials assessed to be of poor quality may have further limited the number and diversity of the included trials. However, the limited number of trials excluded based on the quality assessment suggests that this is unlikely to have had any major impact on our results. Fourth, there might be differences related to the heterogeneity of the included sepsis trials as we included studies with severe sepsis and septic shock defined by either Sepsis-1 or Sepsis-2 criteria and from different parts of the world. Indeed, the degree and type of organ failure in sepsis are shown to be associated with the in-hospital mortality [10]. Unfortunately, heterogeneity is a problem in sepsis and septic shock that seems to be difficult to adjust for. Availability of individual patient-level data can be helpful in this matter. In the meta-analysis by de Grooth and colleagues, a great heterogeneity was found in the control arms of sepsis RCTs, although they only included septic shock patients [55]. This heterogeneity was only partly explained by differences in inclusion criteria and reported baseline characteristic. Interestingly, frequency, distributions and characteristics of the novel sepsis phenotypes identified by Seymour and colleagues were similar in studies with different definitions for sepsis [48]. Due to inconsistency in severity assessment scales and lack of individual patient-level data, we could only adjust for illness severity in subgroup analyses. However, the APACHE II score, which was the most commonly reported score, is an independent risk factor for mortality that contains important components of organ dysfunction [56]. The observation of inconsistency in severity assessment scales highlights the future importance of reporting disease severity by internationally adopted scores such as SOFA and SAPS III.

Finally, studies on trends in mortality from the usual care arm of sepsis trials may shed light on the history of sepsis research and partly mirror the trends in sepsis mortality, but are not suited for sepsis epidemiology surveillance due to the lack of clinical data and heterogeneity issues. The most reliable way to study trends in sepsis mortality seems to be based on clinical raw data derived from electronic healthcare data sets [10]. In a recent paper by Rhee et al., an adjusted version of the SOFA score, the “eSOFA”, optimised for electronic health records, was shown to be of use as a tool in wide-scale surveillance of Sepsis-3 [57]. Hopefully, the initiatives from Centers for Disease Control and Prevention prioritising sepsis as a global health priority will aid governments and healthcare providers to find similar solutions outside the USA in order to the measure the true effects of interventions aiming to reduce the global burden of sepsis.

## Conclusion

Data from RCTs showed a declining trend in 28-day mortality in severe sepsis and septic shock patients during the years from 1991 to 2013. However, when controlling for severity at study inclusion by APACHE II, SAPS II and SOFA scores, there was no significant change in mortality over time. Only 4 of the 44 included trials showed any efficacy with regard to mortality, and none of their methods have been implemented in the current treatment regimen of sepsis. Considering the unsuccessful history of RCTs aiming to lower sepsis mortality, it seems reasonable for future trials to focus on treatments targeted to certain subgroups and to use outcome measures beyond 28-day mortality.

## Additional files

Additional file 1: **Figure S1.** Meta-regression analysis of the temporal trend in Acute Physiology and Chronic Health Evaluation (APACHE) II score between 1991 and 2013 in the included sepsis trials. (DOCX 88 kb)

Additional file 2: **Table S1.** Search strategy. (DOCX 14 kb)

Additional file 3: **Table S2.** Summary of data from trials included in meta-regression analysis. (DOCX 22 kb)

## Data Availability

Available upon request.

## Abbreviations

APACHE: Acute Physiology and Chronic Health Evaluation
PICO: Patient, Intervention, Comparison and Outcome
RCT: Randomised controlled trial
SAPS: Simplified Acute Physiology Score
SOFA: Sequential Organ Failure Assessment
