# Supplementary material for: Trends in sepsis mortality over time in randomised sepsis trials: a systematic literature review and meta-analysis of mortality in the control arm, 2002–2016
Source: Crit Care. 2019 Jul 3;23:241. doi: 10.1186/s13054-019-2528-0 (PMC6610784; doi:10.1186/s13054-019-2528-0)
Supplement: Supplementary file 1 — Figure S1. Meta-regression analysis of the temporal trend in Acute Physiology and Chronic Health Evaluation (APACHE) II score between 1991 and 2013 in the included sepsis trials. (DOCX 88 kb) [file 13054_2019_2528_MOESM1_ESM.docx]

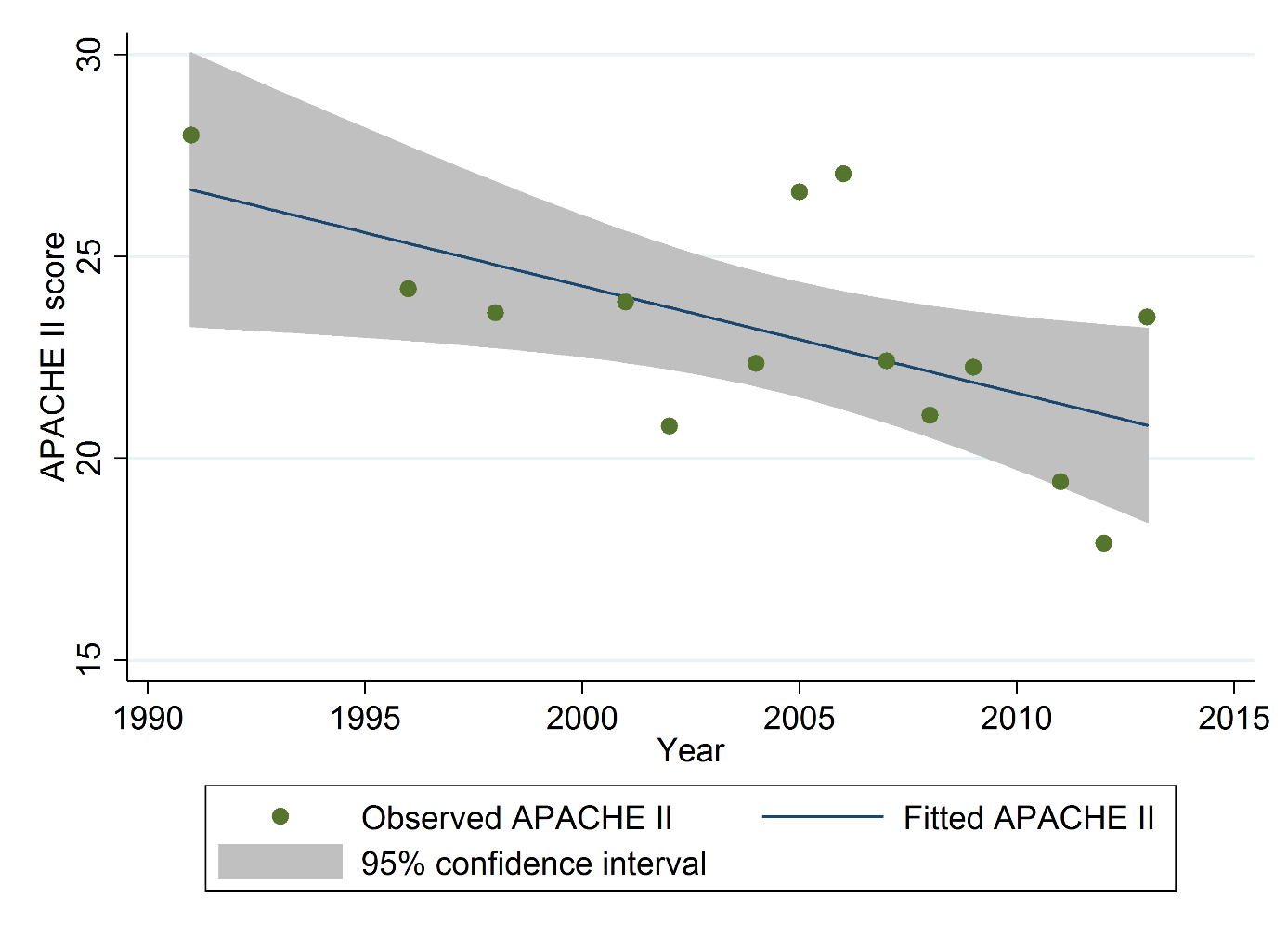


**Figure S1** Meta-regression analysis of the temporal trend in Acute Physiology And Chronic Health Evaluation (APACHE) II score between 1991 and 2013 in the included sepsis trials.
