# Supplementary material for: Trends in sepsis mortality over time in randomised sepsis trials: a systematic literature review and meta-analysis of mortality in the control arm, 2002–2016
Source: Crit Care. 2019 Jul 3;23:241. doi: 10.1186/s13054-019-2528-0 (PMC6610784; doi:10.1186/s13054-019-2528-0)
Supplement: Supplementary file 2 — Table S1. Search strategy. (DOCX 14 kb) [file 13054_2019_2528_MOESM2_ESM.docx]

| Table S1. Search strategy |
| --- |
| (((sepsis[MeSH Terms]) OR ((severe sepsis[Title/Abstract]) OR septic shock[Title/Abstract]))) AND ((mortality[Title/Abstract]) OR “Mortality”[Mesh]) |
| Active filters included: Randomized Controlled Trial; Publication date from 2002/01/01 to 2016/12/31; Humans; English; Adult: 19+ years; Adolescent: 13-18 years |
