# Supplementary material for: Trends in sepsis mortality over time in randomised sepsis trials: a systematic literature review and meta-analysis of mortality in the control arm, 2002–2016
Source: Crit Care. 2019 Jul 3;23:241. doi: 10.1186/s13054-019-2528-0 (PMC6610784; doi:10.1186/s13054-019-2528-0)
Supplement: Supplementary file 3 — Table S2. Summary of data from trials included in meta-regression analysis. (DOCX 22 kb) [file 13054_2019_2528_MOESM3_ESM.docx]

| Table S2. Data from the included studies | | | | | | | | | |
| --- | --- | --- | --- | --- | --- | --- | --- | --- | --- |
| Author | **Mortality usual care** | **Mortality intervention** | **Year of first enrolment** | **Year of publication** | **APACHE* II** | **SAPS ** II** | **SOFA***** | **Age** | **Multinational** |
| Werdan K. et al. | 113/303 | 126/321 | 1991 | 2007 | 28 |  |  | 57,7 | No |
| Root R. et al. | 90/353 | 101/348 | 1996 | 2003 | 24 |  |  | 60 | Yes |
| Abraham E. et al. | 65/196 | 143/390 | 1998 | 2003 | 23,6 |  |  | 57,3 | No |
| Annane D. et al. | 64/161 | 54/169 | 1999 | 2007 |  | 54 | 11 | 65 | No |
| Angstwurm M. et al. | 55/97 | 39/92 | 1999 | 2007 |  |  |  | 65,9 | No |
| Opal S. et al | 150/617 | 160/639 | 2001 | 2004 | 21,8 |  |  | 60,1 | Yes |
| Zeiher B. et al. | 59/185 | 74/188 | 2001 | 2005 | 24,1 |  |  | 60,8 | N/A |
| Russel J. et al. | 150/382 | 140/369 | 2001 | 2008 | 27,1 |  |  | 61,8 | Yes |
| Abraham E. et al. | 220/1297 | 243/1316 | 2002 | 2005 | 18,2 |  |  | 58,6 | Yes |
| Levi M. et al. | 305/955 | 275/972 | 2002 | 2007 | 24 |  |  | 58,4 | Yes |
| Sprung C. et al. | 78/248 | 86/251 | 2002 | 2008 |  | 48,6 | 10,6 | 63 | Yes |
| Tidswell M. et al. | 27/78 | 26/80 | 2002 | 2010 | 24,9 |  |  | 60,6 | Yes |
| Myburgh J. et al. | 36/138 | 31/138 | 2004 | 2008 | 22,2 |  |  | 60,4 | No |
| Dellinger P. et al. | 161/599 | 154/598 | 2004 | 2009 | 21,5 |  |  | 63,1 | Yes |
| Dhainaut JF. et al. | 31/99 | 37/94 | 2004 | 2009 | 27,7 |  |  | 62,7 | Yes |
| Rice T. et al. | 22/91 | 16/92 | 2005 | 2010 | 26,6 |  | 8,3 | 60,6 | Yes |
| Joannes-Boyau O. et al. | 29/71 | 25/66 | 2005 | 2013 |  | 64 | 12 | 70 | Yes |
| Shorr A. et al. | 36/224 | 51/205 | 2006 | 2010 | 26,3 |  | 8,4 | 62,3 | Yes |
| COIITSS Stud. Inv. | 99/254 | 96/255 | 2006 | 2010 |  | 60,4 | 10,8 | 64,3 | No |
| Opal S. et al. | 177/657 | 366/1304 | 2006 | 2013 | 27,3 |  |  | 65,8 | Yes |
| Brunkhorst F. et al. | 59/269 | 66/276 | 2007 | 2012 | 21,9 |  | 9,7 | 63,7 | No |
| Guidet B. et al. | 24/95 | 31/100 | 2007 | 2012 |  | 53 | 9,1 | 65,9 | Yes |
| Kruger P. et al. | 22/127 | 12/123 | 2007 | 2013 | 23,5 |  | 8 | 64 | Yes |
| Livigni S. et al. | 28/93 | 37/91 | 2007 | 2014 |  | 53 | 9 | 64,9 | No |
| Ranieri M. et al. | 202/834 | 223/846 | 2008 | 2012 | 25,5 |  |  | 62,7 | Yes |
| ARISE Inv. | 127/797 | 117/792 | 2008 | 2014 | 15,8 |  |  | 63,1 | Yes |
| Annane D. et al. | 70/203 | 76/208 | 2008 | 2013 |  | 55 | 10 | 64 | No |
| Wu J. et al. | 63/180 | 47/181 | 2008 | 2013 | 21,6 |  | 7,7 | 66,4 | No |
| Guntupalli K. et al. | 25/93 | 14/97 | 2008 | 2013 | 25,4 |  | 9 | 61 | No |
| Hyvernat H. et al. | 31/59 | 28/63 | 2008 | 2016 |  | 57,9 | 10,4 | 64,3 | N/A |
| Caironi P. et al. | 288/900 | 285/895 | 2008 | 2014 |  | 48 | 8 | 69 | No |
| Karnad D. et al. | 12/59 | 4/55 | 2009 | 2014 | 13,5 |  |  | 36,7 | No |
| Keh D. et al. | 14/170 | 15/171 | 2009 | 2016 | 18,5 | 52,2 | 6,2 | 64,6 | No |
| Bloos F. et al. | 60/262 | 89/267 | 2009 | 2016 | 24,4 |  | 9,9 | 65,6 | No |
| Perner A. et al. | 144/400 | 154/398 | 2009 | 2012 |  | 51 | 7 | 67 | Yes |
| Morelli A. et al. | 62/77 | 28/77 | 2010 | 2013 |  | 57 |  | 69 | No |
| Asfar P. et al. | 132/388 | 142/388 | 2010 | 2014 |  | 57,2 | 10,8 | 65 | No |
| Bernard G. et al. | 20/99 | 15/100 | 2010 | 2014 |  |  |  |  | No |
| Payen D. et al. | 22/113 | 33/119 | 2010 | 2015 |  | 59 | 10 | 72 | No |
| Vincent JL. et al. | 27/152 | 38/153 | 2011 | 2015 | 25,2 |  | 8,9 | 61,6 | Yes |
| Mouncey P. et al. | 152/621 | 155/625 | 2011 | 2015 | 18 |  | 4,3 | 64,3 | No |
| Andrews B. et al. | 36/54 | 35/49 | 2012 | 2014 | 17,9 |  |  | 34,8 | No |
| Kuan W. S. et al. | 6/61 | 8/61 | 2012 | 2015 |  |  | 3,2 | 64,3 | No |
| Gordon A. et al. | 56/204 | 63/204 | 2013 | 2016 | 24 |  | 6,2 | 63 | No |
| *Acute Physiology And Chronic Health Evaluation ** Simplified Acute Physiology Score  *** Sequential Organ Failure Assessment | | | | | | | | | |
